# International experience of hikikomori (prolonged social withdrawal) and its relevance to psychiatric research

**DOI:** 10.1192/bji.2020.20

**Published:** 2021-05

**Authors:** Marcus P. J. Tan, William Lee, Takahiro A. Kato

**Affiliations:** 1 ST5 Trainee in Child and Adolescent Psychiatry, South London and Maudsley NHS Foundation Trust, UK. Email: MPJTan@doctors.org.uk; 2 Consultant Liaison Psychiatrist, Devon Partnership NHS Trust, Exeter, UK; 3 Associate Professor, Department of Neuropsychiatry, Graduate School of Medical Sciences, Kyushu University, Japan

**Keywords:** Aetiology, transcultural psychiatry, social functioning

## Abstract

When prolonged social withdrawal was first described in Japan as ‘hikikomori’, many studies examining its etiology suggested it to be related to factors unique to Japan and thus a culture-bound syndrome. However, existing research has suffered from a lack of standardised definitions, impeding comparability between studies. We summarise existing research and discuss its relevance to psychiatric practice today.

## What is hikikomori?

In Japan, there exists the term ‘hikikomori’, used to refer to a particularly severe form of social withdrawal. The term is part of the everyday lexicon in the Japanese language, where it is a compound verb made up of the two characters for ‘to pull back’ (*hiku*) and ‘to seclude oneself’ (*komoru*), and is used to describe a person's deliberate seclusion of themselves from mainstream society.^1^ The term was introduced to the medical community by the Japanese psychiatrist Tamaki Saito in 1998 in a bestselling book, where he used it as a noun to describe typically young persons who have stopped going to school or work, instead withdrawing into their homes for remarkably long periods.^2^ The term has also entered the English language (including an entry in the Oxford English Dictionary), where it is used to refer to both the condition and the individual. We mirror those usages here. Saito's text was highly regarded for describing a hitherto unrecognised problem that had its roots in Japanese history and culture, dating back to ‘futouko’ (school refusal) in the 1970s. Subsequently a prevalence study by Koyama et al using a nationally representative sample of Japan suggested that 1.2% of 20- to 49-year-olds had experienced this condition.^3^ The concern about its growing prevalence, as well as its potential impact on an individual's mental health and wider society, make hikikomori an urgent issue for the Ministry of Health, Labour and Welfare in Japan.

## Defining hikikomori

Despite extensive research on hikikomori over the past two decades, studies rarely utilise a standardised definition for it. A systematic review in 2015^4^ highlights three areas in which the operational definition of hikikomori can differ:

• social connectedness: while researchers generally agree that individuals with hikikomori are detached from the social structure, there is disagreement over whether having or maintaining personal relationships with a few significant others should be considered an exclusion criterion;
• place of withdrawal: there is disagreement over the extent to which the definition is limited only to people confined to their home, or whether those who infrequently leave their home may also be included;
• duration of withdrawal: traditionally, hikikomori has been defined as social withdrawal for 6 months or more; however, some advocate a shorter duration (i.e. 3 months) as also being problematic.

In 2020, Kato, Kanba and Teo proposed diagnostic criteria for hikikomori that addressed this to some degree by classifying hikikomori who infrequently leave their home on a spectrum of severity, and classifying those who do not yet meet the 6-month criterion as ‘pre-hikikomori’ (Box 1).^5^ However, the criteria do not specify the extent of the lack of social connectedness required for diagnosis. In particular, online relationships are a possible avenue through which hikikomori remain socially connected despite their physical isolation, highlighting the need for assessment and diagnostic tools for hikikomori that are updated and relevant.

Box 1 Diagnostic criteria of hikikomori (pathological social withdrawal) proposed by Kato et al^5^

Hikikomori is a form of pathological social withdrawal or social isolation whose essential feature is physical isolation in the person's home. Three accompanying criteria are proposed:

(a) marked social isolation in the person's home
(b) duration of continuous social isolation of at least 6 months
(c) significant functional impairment or distress associated with the social isolation.

Individuals who occasionally leave their home (on 2 or 3 days/week), rarely leave their home (1 day/week or less) or rarely leave a single room may be characterised as having mild, moderate or severe disorder respectively. Individuals who leave their home frequently (on 4 or more days/week), by definition, do not meet criteria for hikikomori.

The estimated continuous duration of social withdrawal should be noted. Individuals with a duration of at least 3 (but not 6) months of social isolation should be classified as pre-hikikomori.

Hikikomori tends to co-occur with other psychiatric conditions, and comorbidity with other psychiatric disorders is not as an exclusion criterion.

## Hikikomori and other psychiatric disorders

The extent to which hikikomori may overlap with existing psychiatric conditions is unclear. Several studies have attempted to examine this, but they have been inconsistent in screening for the full range of psychiatric disorders. For example, a prevalence study of hikikomori in Japan by Koyama et al reported that 45.5% of hikikomori did not have a lifetime prevalence of another psychiatric disorder.^3^ One strength of the study is its used of a standardised instrument to screen for comorbidity – the World Health Organization Composite International Diagnostic Interview. However, personality disorders, psychotic disorders and neurodevelopmental disorders were not considered in the screening process. A Ukrainian study, which recruited in-patients and out-patients with hikikomori from a tertiary hospital, found that 13 of their 35 participants did not have a comorbid diagnosed mental disorder; however, only mental disorders in the ICD-10 category of ‘neurotic, stress-related and somatoform disorders’ (F40–48) were considered.^6^ On the other hand, a Japanese study examining 337 hikikomori referrals found that only one did not have a comorbid psychiatric diagnosis,^7^ and a Spanish study that followed up 190 individuals with hikikomori also found only one person without associated psychopathology.^8^ Thus, studies that screened for the full range of psychiatric disorders found that the majority of hikikomori also had a comorbid mental disorder.

Given that hikikomori tends to exist with other psychiatric disorders, does this mean that it is wholly explainable using current diagnostic frameworks? Indeed, guidelines published by the Japanese Ministry of Health, Labour and Welfare in 2010 stated that, while hikikomori ‘in general […] is considered to be a non-psychotic phenomenon […] it is not unlikely that cases may include schizophrenia before a definitive diagnosis is reached’. Such claims add support to the suggestion that hikikomori may be a manifestation of conventional psychiatric disorder, with the withdrawal merely being a contributor to a more overarching psychopathology.^9^ Nevertheless, there remain several hikikomori cases that present with severe and disabling levels of social withdrawal, yet do not fit classification into any known diagnostic framework. That said, such evidence is scant, and the use of varying definitions of hikikomori across studies have made it difficult to draw firm conclusions. To this end, it is hoped that the new diagnostic criteria set out in Box 1 will help standardise definitions of hikikomori and encourage more robust comparisons to be made.^5^ These criteria have also removed the presence of other psychiatric disorder as an exclusion criterion, and it is hoped that future research will add to existing knowledge on hikikomori and its possible relationship with existing psychopathology.

## Cultural perspectives on hikikomori

There has been extensive research on hikikomori outside of Japan. In addition to the above-mentioned studies from Spain^8^ and Ukraine,^6^ there are also reports of cases in Hong Kong, Oman, France, Italy and the USA.^1,10^ In addition, an international survey using a case vignette description of hikikomori suggested that psychiatrists from Australia, Bangladesh, Iran, India, Japan, South Korea, Taiwan, Thailand and the USA all felt that they had dealt with such phenomena in their clinical practice.^11^ There are also prevalence studies conducted in Hong Kong^7^ and China.^12^

The identification of cases in countries outside Japan challenges previous narratives of hikikomori as a culture-bound syndrome, best understood and managed primarily from the cultural perspective from which it originates.^9^ However, there is increasing evidence that aspects of the Japanese sociocultural environment felt to be essential in hikikomori aetiology may not be ‘culture-bound’ to Japan. The most extensively studied of these aspects in the Japanese context is ‘amae’: an overdependent behaviour, described as a feature of Japanese parent–child relationships by psychoanalyst Takeo Doi. This has been hypothesised to play a role in developing hikikomori by normalising and encouraging the acceptance of social withdrawal behaviour and consequently allowing sufferers to stay at home for long periods.^1^ However, similarities between amae and other parenting styles in East Asian countries have also been found.^13^ Other examples include ‘haji’ (shame) and ‘ijime’ (bullying), where they are conceptualised as stressors that could precipitate hikikomori or hikikomori-like behaviour.^1^ However, how such Japanese cultural factors relate to the wider sociocultural environment – and indeed how precisely they lead to the eventual outcome of social withdrawal – is poorly understood. While the direct translations of haji and ijime may suggest that they refer to concepts that are shared by non-Japanese speakers, it may be a mistake to assume that their experience of it is similar. For example, it is possible that there are specific sociocultural aspects of Japanese society, such as a collectivistic mindset that encourages exclusion of outsiders, that make the experience of these more traumatic. Further research is needed to study whether such differences are significant in the development of hikikomori across cultures.

## Conclusions

As a condition, hikikomori stands out owing to the extreme levels of isolation and associated impairment. Existing research suggests that, while many hikikomori have additional psychiatric conditions, there remains a small but significant proportion who do not seem to meet criteria for any other current diagnostic category. Hikikomori may also be a useful construct in the study of social withdrawal within other psychiatric conditions. Further work should explore this relationship in detail, utilising standardised diagnostic measures such as semistructured interviews or standardised instruments such as the recently published 25-item Hikikomori Questionnaire.^14^ Finally, research could also investigate potential overlaps with related concepts, such as loneliness and social isolation. Attempting a synthesis of knowledge on these would help reveal all aspects of these important conditions.
